# Total Shoulder Arthroplasty Versus Reverse Shoulder Arthroplasty in Primary Glenohumeral Osteoarthritis With Intact Rotator Cuffs: A Meta-Analyses

**DOI:** 10.7759/cureus.57866

**Published:** 2024-04-08

**Authors:** Neeraj Vij, Sailesh Tummala, Eahsan Shahriary, John Tokish, Shelden Martin

**Affiliations:** 1 Department of Orthopedic Surgery, University of Kansas School of Medicine-Wichita, Wichita, USA; 2 Department of Orthopedic Surgery, Mayo Clinic, Phoenix, USA; 3 School of Public Health, University of California, Berkeley, Berkeley, USA; 4 Department of Orthopedic Surgery, OrthoArizona, Phoenix, USA

**Keywords:** evidence-based practice, surgical decision-making, glenohumeral arthritis, shoulder surgery, surgical outcomes, degenerative joint disease, shoulder replacement

## Abstract

Traditional practice favors total shoulder arthroplasty (TSA) for the treatment of primary glenohumeral osteoarthritis (PGHO) with an intact rotator cuff; however, the indications for reverse shoulder arthroplasty (RSA) have expanded to include PGHO. The purpose of this systematic review is to compare the mean differences in the range of motion and patient-reported outcomes between the TSA and RSA with an intact rotator cuff and to analyze the subgroup of the Walch type B2 glenoid. This IRB-exempt, PROSPERO-registered systematic review strictly followed the Preferred Reporting Items for Systematic Reviews and Meta-Analysis Protocols (PRISMA-P) guidelines. A literature search of five databases revealed 493 articles, of which 10 were included for quantitative synthesis. Level III evidence studies with the diagnosis of PGHO and ≥2 years of follow-up were included. Studies without preoperative and postoperative data were excluded. The Newcastle-Ottawa scale was used to evaluate the methodologic quality of the included studies. Preoperative and postoperative range of motion and patient-reported outcomes were collected. The random-effects model was employed, and p < 0.05 was considered statistically significant. There were a total of 544 and 329 studies in the TSA group and RSA group, respectively. The mean age in the TSA group and RSA groups were 65.36 ± 7.06 and 73.12 ± 2.40, respectively (p = 0.008). The percentages of males in the TSA and RSA groups were 73.2% and 51.1%, respectively (p = 0.02). The mean differences in forward elevation, external rotation in adduction, internal rotation scale, visual analog scale (VAS), American Shoulder and Elbow Surgeons (ASES) score, and Single Assessment Numeric Evaluation (SANE) scores were improved for both groups with no significant differences between the two. There were 9.6 times the revisions in the TSA group (8.8% vs. 0.91%; p = 0.014) and 1.5 times the complications in the TSA group (3.68% vs. 2.4%; p = 0.0096). Two hundred and forty-two glenoids were identified as Walch type B2 (126 in the TSA group and 116 in the RSA group). The mean ages in the B2 subgroup were 68.20 ± 3.25 and 73.03 ± 1.49 for the TSA and RSA, respectively (p = 0.25). The percentages of males in the B2 subgroup were 74.6% and 46.5% for the TSA and RSA groups, respectively (p = 0.0003). The ASES, SANE, forward elevation, and external rotation in the adduction results were descriptively summarized for this subgroup, with average mean differences of 49.0 and 51.2, 45.7 and 66.1, 77.6° and 58.6°, and 38.6° and 34.1° for the TSA and RSA groups, respectively. In the setting of primary glenohumeral osteoarthritis with an intact rotator cuff, the RSA has a similar range of motion and clinical outcomes but lower complication and revision rates as compared to the TSA. This may hold true in the setting of the B2 glenoid, although a high-powered study on this subgroup is required. Anatomic shoulder arthroplasty maintains an important role in select patients. Further studies are required to better elucidate the role of glenoid bone loss and posterior humeral head subluxation with regard to implant choice.

## Introduction and background

The indications for reverse shoulder arthroplasty (RSA) have grown substantially over the past few decades to include glenohumeral arthritis with significant glenoid bone deformity [1], irreparable rotator cuff tear without arthropathy [2], and complex proximal humerus fractures [3]. RSA is being performed at increasingly higher rates in the setting of primary glenohumeral osteoarthritis (PGHO) in elderly patients [2]. The number of RSAs performed yearly in the United States has surpassed the number of total shoulder arthroplasties (TSAs) both overall and for PGHO [4].

There is controversy in the setting of PGHO (intact rotator cuff) as to whether the TSA or RSA results in superior clinical outcomes. Proponents of the RSA cite the versatility and reproducibility of this procedure. A recent systematic review demonstrated that functional and patient-reported outcomes in RSA do not vary significantly based on the preoperative diagnosis [5]. However, historically, TSA has been the gold standard for patients with PGHO and an intact rotator cuff, given the excellent outcomes associated with restoring native biomechanics.

Glenoid wear is an important consideration in the evaluation of the patient. Walch et al. [6] described three glenoid wear patterns that have subsequently been expanded upon: A or concentric wear, B or posterior/eccentric wear, and C or dysplastic glenoids. B glenoids can be further described as B1 or monoconcave, B2 or biconcave glenoids, and B3 or monoconcave glenoids with posterior subluxation >70% or retroversion > 15% [7]. Glenoid deformity can influence treatment decisions with neoglenoid retroversion of greater than 27 degrees leading to an increased risk of glenoid component loosening [8]. However, the type B2 glenoid remains an indeterminate area where the decision between an RSA and TSA can be difficult [9]. The degree of bone deformities and surgeon comfort regarding handling glenoid eccentricity can influence this surgical decision [8,10,11]. However, the evidence to assist surgeons in making this clinical decision is limited. The purpose of this meta-analysis is to compare the mean differences between preoperative and postoperative range of motion and patient-reported outcomes between TSA and RSA with an intact rotator cuff and to analyze the subgroup of the Walch type B2 glenoid.

## Review

Materials and methods

General

This IRB-exempt meta-analysis was registered with the International Prospective Register of Systematic 3.1.3 Patient-Reported Outcomes Reviews (PROSPERO No. CRD42022297549). The Preferred Reporting Items for Systematic Review and Meta-Analysis Protocols (PRISMA-P) guidelines were followed.

Search Strategy

A literature search was performed using the following keywords: Population: “primary glenohumeral osteoarthritis” OR “primary glenohumeral arthritis”; Intervention: “reverse shoulder arthroplasty” OR “RSA” OR “Grammont style replacement” AND "total shoulder arthroplasty" OR "Anatomic Shoulder Arthroplasty"; Outcomes: “patient-reported outcomes” OR “clinical outcomes” OR “functional outcomes” OR “radiographic outcomes”. PubMed, Cochrane, Web of Science Collection, Scopus, Google Scholar, MEDLINE, CINAHL, and Embase databases were searched. Search fields were varied until no new articles were collected at which point the search was considered exhaustive.

Study Screening and Selection

The search yielded 493 studies. Based on the title and abstract review, a total of 40 article abstracts were selected for review. Full-text screening of these articles was performed based on our inclusion/exclusion criteria (Table 1).

**Table 1:** Our inclusion and exclusion criteria as applied independently by three of the authors during the initial title/abstract review. VAS: visual analog scale, ASES: American Shoulder and Elbow Surgeons, WOOS: Western Ontario Osteoarthritis of the Shoulder, SST: Simple Shoulder Test, SANE: Single Assessment Numeric Evaluation, DASH: Disabilities of the Arm, Shoulder, and Hand Questionnaire, SF-12 PCS: 12-item Short Form Health Survey Physical Composite Scale

Inclusion criteria	Exclusion criteria
Level III evidence or higher	Systematic reviews, review papers, case reports, case series, and other level IV/level V studies
Ages 18-90	Absence of preoperative and postoperative data
Diagnosis of primary glenohumeral osteoarthritis	Hemiarthroplasty, head replacement surgeries/stemless implants, recessed glenoid components
Follow-up of ≥2 years	Prior existing rotator cuff disease
Presence of any of the following outcome measures: VAS scores, satisfaction scores, ASES scores, WOOS scores, SST scores, constant-total scores, SANE scores, Quick DASH scores, SF-12 PCS scores, revision rate, complication rate, or postoperative range of motion	Prior shoulder arthroplasty

**Figure 1:**
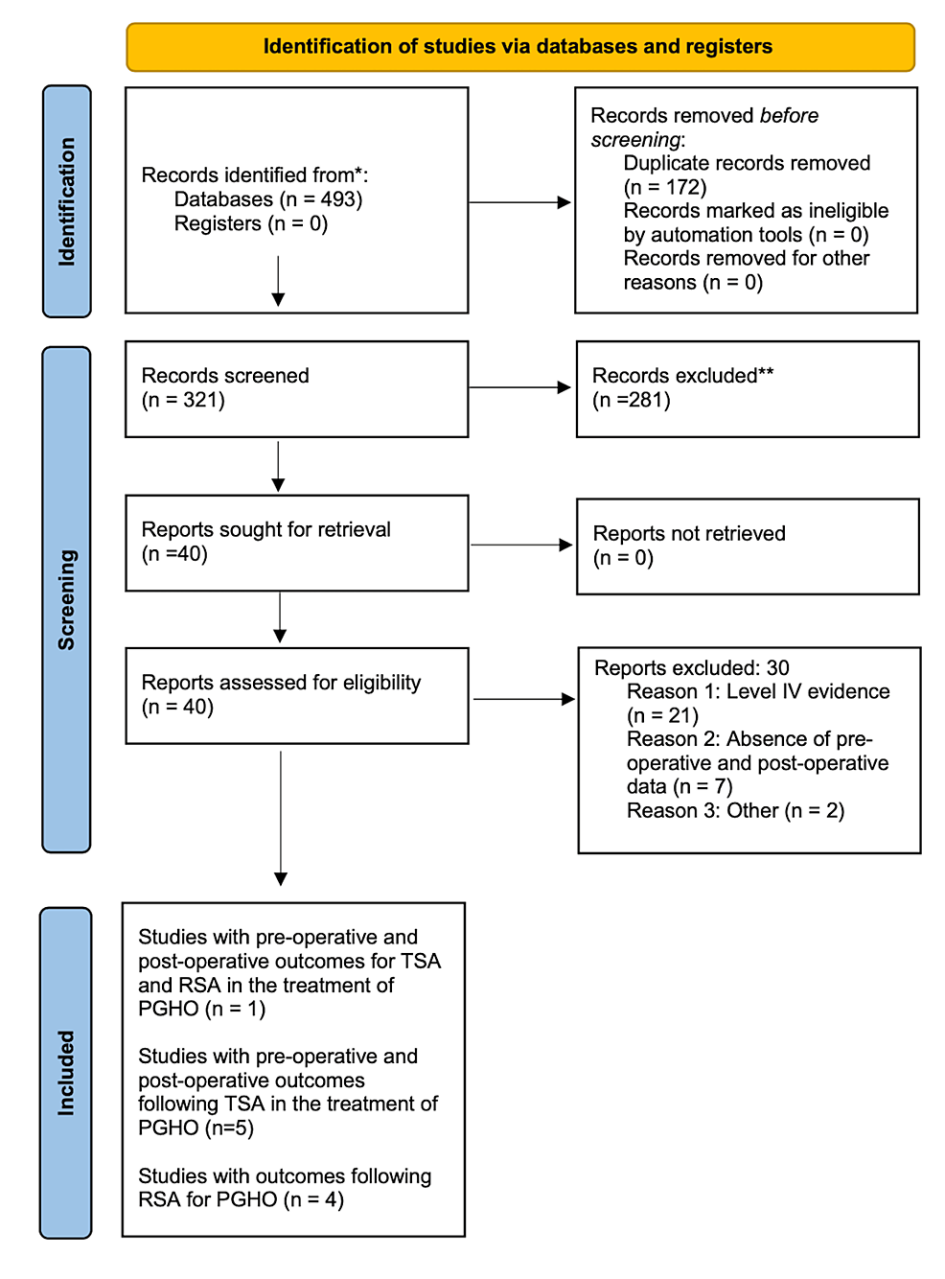
The Preferred Reporting Items for Systematic reviews and Meta-Analyses (PRISMA) flow diagram for our study.

The references in these articles were also hand-searched to identify any missing articles. This resulted in 10 articles included in the quantitative synthesis articles (Figure 1).

Quality Assessment

To assess the included studies, guidelines from the Revised Assessment of Multiple SysTemAtic Reviews (R-AMSTAR) were followed [12]. The Newcastle-Ottawa scale was chosen considering that all included studies were cohort studies. Studies were evaluated based on the selection, comparability, and outcome criteria.

Data Collection

The data elements extracted from the articles fell into the categories of study basics, demographic data, glenoid type (when available), physical exam metrics, patient-reported outcomes, and complications. The variables under study basics included lead author, year published, journal of publication, study type, level of evidence, the risk of bias as determined above, and length of follow-up. The variables under demographic information were age, gender, date of publication, and country of origin of the publication. The physical examination metrics collected included forward elevation, abduction, external rotation in 90 of abduction (ER in Abd) external rotation in adduction (ER in Add), and internal rotation (IR). The variables under patient-reported outcomes included Visual Analog Scale (VAS) scores, Satisfaction Scores, American Shoulder and Elbow (ASES) Scores, Western Ontario Osteoarthritis of the Shoulder (WOOS) Scores, Simple Shoulder Test (SST) Scores, Constant-Murley (CM) Total Scores, Single Assessment Numeric Evaluation (SANE) scores, Quick DASH scores, and 12-item Short Form Health Survey Physical Composite Scale (SF-12 PCS) Scores. All documented complications and the duration of follow-up were also recorded.

Data Abstraction and Statistical Analysis

The data analysis was performed by a statistician at the University of Arizona Department of Biostatistics. Frequencies and proportions were calculated for categorical variables. Means and standard deviations were calculated for continuous variables. Data abstraction and figure creation were performed with Microsoft Excel V16.42. A two-sample T-test was used to compare the preoperative values for both range of motion and patient-reported outcomes. The random-effects model (REML) was used to compare the mean differences for the range of motion and patient-reported outcomes. The Mann-Whitney test was used to compare revision rates and complication rates. The I_2_ statistic, which explains the percentage of variation across studies that is due to heterogeneity rather than chance and should be included in all systematic reviews, was also calculated [13]. Forest plots were chosen to represent the data considering the homogeneity in reported outcomes between the ≥2 included studies for any given outcome measure. All two-tailed P values <0.05 were considered statistically significant. P-values were two-tailed with P < 0.05 considered statistically significant. Stata Statistical Software (release 17, StataCorp LLC, College Station, TX, 2021) was used for all statistical analyses.

Results

General

There were a total of 10 studies that met our inclusion and exclusion criteria (Table 2).

**Table 2:** A summary of the primary literature included in our study. *This study contained 15 patients who were included in the total shoulder arthroplasty (TSA) group and 16 patients who were included in the reverse shoulder arthroplasty (RSA) group. PGHO: primary glenohumeral osteoarthritis, TSA: total shoulder arthroplasty, RSA: reverse shoulder arthroplasty

TSA and RSA, TSA, or RSA	Lead author last name	Year published	Journal	Study type	Level of evidence	PGHO shoulder number (intact cuff)	Walch type B2 shoulder number
TSA and RSA	Alentorn-Geli [9]	2018	Journal of Orthopaedic Surgery	Retrospective cohort study	III	31*	31
TSA	Petri [14]	2016	Archives of Orthopedic Traumatology and Surgery	Retrospective cohort study	III	78	Could not determine
TSA	Sheth [15]	2020	Journal of Shoulder and Elbow Surgery	Prospective observational study	II	111	111
TSA	Neyton [16]	2019	Journal of Shoulder and Elbow Surgery	Retrospective cohort study	III	155	Could not determine
TSA	Hussey [17]	2015	Journal of Shoulder and Elbow Surgery	Retrospective cohort study	III	148	Could not determine
TSA	Nolte [18]	2021	The American Journal of Sports Medicine	Retrospective cohort study	III	37	Could not determine
RSA	Waterman [19]	2020	Journal of the American Academy of Orthopaedic Surgeons	Retrospective cohort study	III	43	43
RSA	Pettit [20]	2021	Journal of Shoulder and Elbow Surgery	Retrospective cohort study	III	116	57
RSA	Lindbloom [21]	2019	Journal of Shoulder and Elbow Surgery	Retrospective cohort study	III	129	Could not determine
RSA	Wall [22]	2007	The Journal of Bone and Joint Surgery	Prospective cohort observational study	II	25	Could not determine

One study directly compared the outcomes of RSA and TSA [9], five studies provided outcomes after TSA for the treatment of PGHO [14-18], and four studies provided outcomes after RSA for the treatment of PGHO [19-22]. The results of our methodologic quality analysis with the Newcastle-Ottawa scale are provided in Table 3.

**Table 3:** Results of grading the clinical evidence using the Newcastle-Ottawa Scale from our included studies.

Author last name	Selection	Comparability	Outcome
Alentorn-Geli [9]	****	**	***
Petri [14]	****	**	***
Sheth [15]	***	**	***
Neyton [16]	****	**	**
Hussey [17]	****	**	***
Nolte [18]	****	*	***
Waterman [19]	****	**	***
Pettit [20]	***	**	***
Lindbloom [21]	****	**	**
Wall [22]	****	**	***

There were a total of 544 shoulders in our TSA group and 329 shoulders in our RSA group. Of these, 301 could be identified by Walch glenoid type, which included 10 B1 glenoids, 242 B2 glenoids, and 49 B3 glenoids. The mean ages in the TSA group and RSA groups were 65.36 ± 7.06 and 73.12 ± 2.40, respectively (P = 0.008). The percentages of males in the TSA and RSA groups were 73.2% and 51.1%, respectively (P = 0.02). There were no statistically significant differences between the TSA and RSA groups in terms of preoperative range of motion or patient-reported outcomes (Table 4).

**Table 4:** Comparison between our TSA and RSA groups preoperatively. TSA: total shoulder arthroplasty, RSA: reverse shoulder arthroplasty, ER in Add: external rotation in adduction, VAS: visual analog scale, ASES: American Shoulder and Elbow Society, SANE: Single Assessment Numeric Evaluation

Variable	TSA	RSA	P-Value
Forward elevation	95.95° (8.37°)	84.48° (6.96°)	0.15
ER in add	13.93° (5.82°)	21.94° (9.35°)	0.21
Internal rotation	2.8 (0.85)	2.74 (0.37)	0.94
VAS - pain	5.00 (1.41)	4.24 (1.73)	0.64
ASES	44.58 (4.61)	38.78 (8.03)	0.38
SANE	44.4 (12.5)	30.37 (0.467)	0.19

Physical Examination

**Figure 2:**
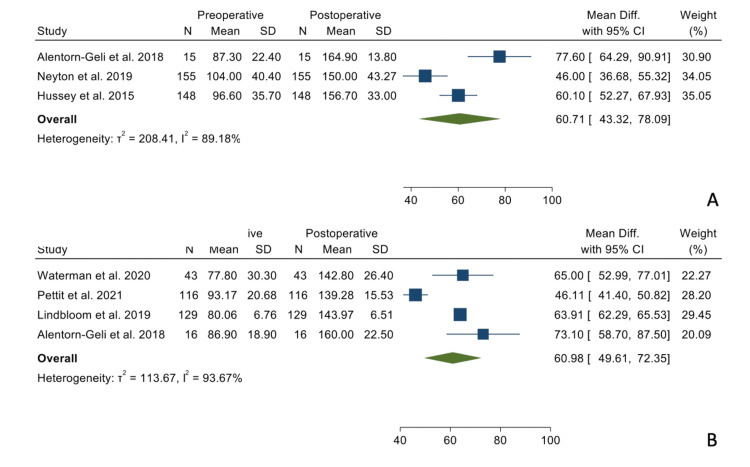
Mean differences in forward elevation between the TSA group (Panel A) and the RSA group (Panel B). References: [9,16,17,19-21]

**Figure 3:**
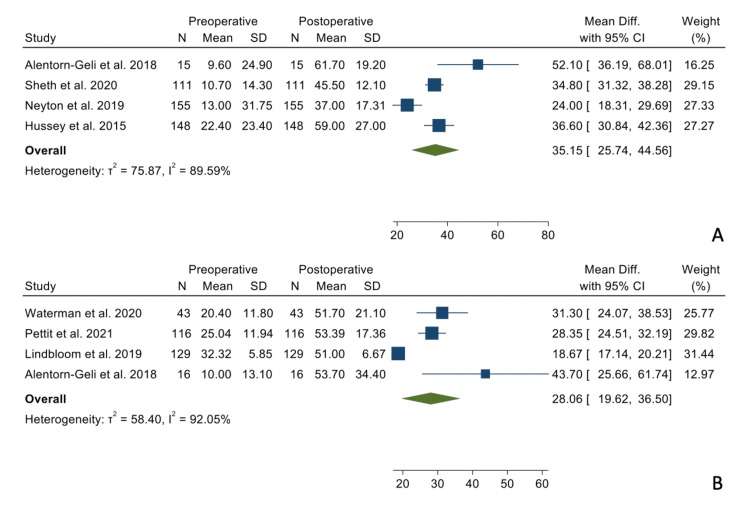
Mean differences in external rotation in adduction in the TSA group (Panel A) and the RSA group (Panel B). References: [9,15-17,19-21]

**Figure 4:**
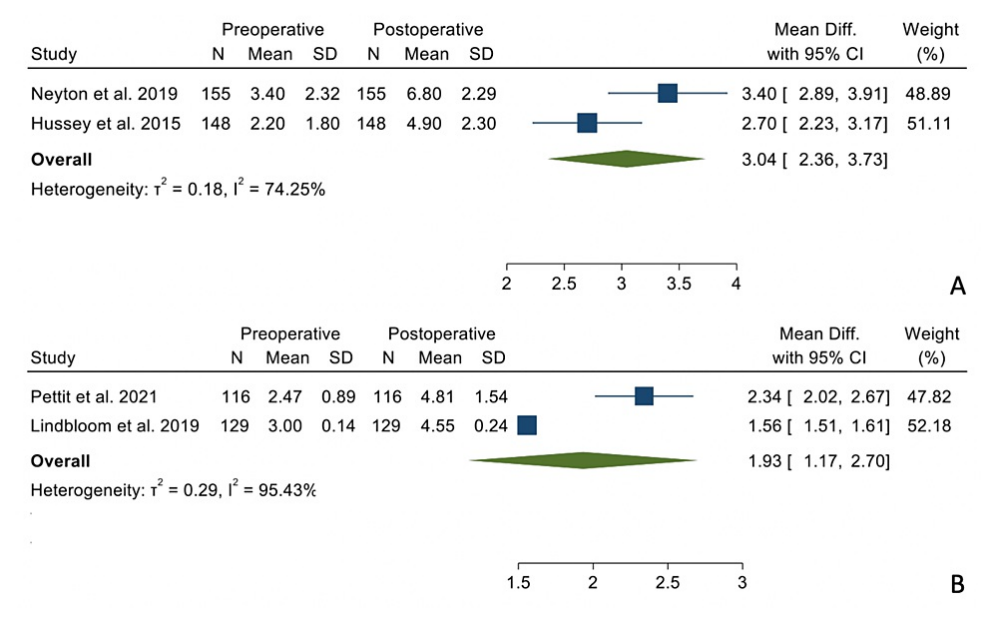
Mean differences in internal rotation in the TSA (Panel A) and the RSA group (Panel B). References: [16,17,20,21]

There were no statistically significant differences when comparing the mean differences between the preoperative and postoperative states between the two groups regarding forward elevation (Figure 2), external rotation in adduction (Figure 3), and internal rotation (Figure 4).

The summary data regarding mean differences between preoperative and postoperative data can be seen in Table 5.

**Table 5:** Summary of the mean differences between the preoperative and postoperative states for all outcomes of our study. TSA: total shoulder arthroplasty, RSA: reverse shoulder arthroplasty, ER in Add: external rotation in adduction, VAS: visual analog scale, ASES: American Shoulder and Elbow Society, SANE: Single Assessment Numeric Evaluation

	TSA		RSA		
Variable	Mean difference	Minimum, maximum	Mean difference	Minimum, maximum	p-value
Forward elevation	60.71°	43.32°, 78.09°	60.98°	49.61°, 72.35°	0.883
ER in Add	35.15°	25.74°, 44.56°	28.06°	19.62°, 36.50°	0.197
Internal rotation	3.04	2.36, 3.73	1.93	1.17, 2.70	0.308
VAS - pain	-3.93	-4.26, -3.60	-4.65	-6.21, -3.08	0.657
ASES	42.62	37.29, 48.00	44.59	35.25, 53.92	0.71
SANE	40.34	33.18, 47.51	58.38	52.26, 64.49	0.095

Patient-Reported Outcomes

**Figure 5:**
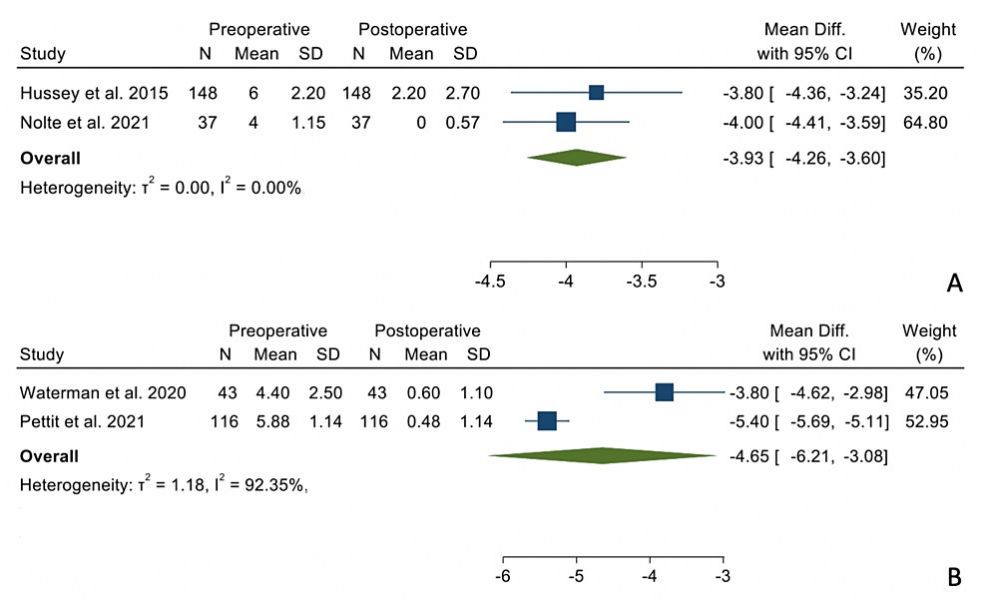
Mean differences in VAS-Pain scores in the TSA group (Panel A) and the RSA group (Panel B). VAS: visual analog scale, TSA: total shoulder arthroplasty, RSA: reverse shoulder arthroplasty References: [17-20]

**Figure 6:**
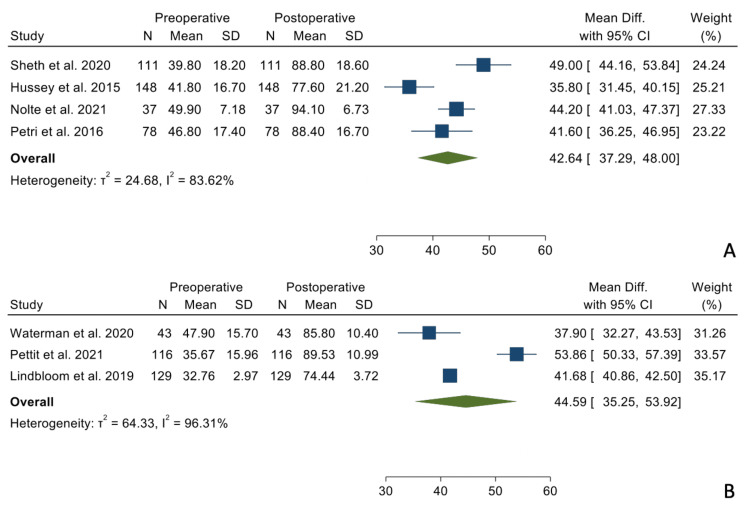
Mean differences in ASES scores in the TSA group (Panel A) and the RSA group (Panel B). ASES: American Shoulder and Elbow Surgeons, TSA: total shoulder arthroplasty, RSA: reverse shoulder arthroplasty References: [14,15,17-21]

**Figure 7:**
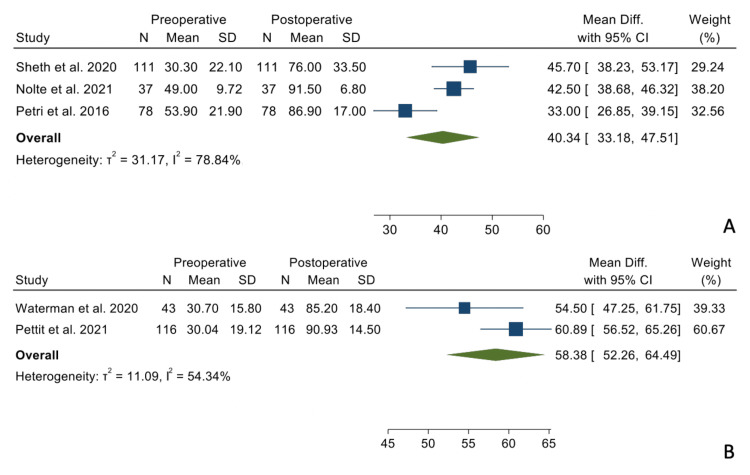
Mean differences in SANE scores in the TSA group (Panel A) and the RSA group (Panel B). SANE: Single Assessment Numeric Evaluation, TSA: total shoulder arthroplasty, RSA: reverse shoulder arthroplasty References: [14,15,18-20]

There were no statistically significant differences when comparing the mean differences between the preoperative and postoperative states between the two groups regarding VAS-Pain scores (Figure 5), ASES scores (Figure 6), and SANE scores (Figure 7).

The summary data regarding mean differences between preoperative and postoperative data can be seen in Table 5.

Revisions

There were a total of 48 (8.8%) revisions in the TSA group and three revisions (0.91%) in the RSA group (P = 0.014).

Complications

There were a total of 20 (3.68%) complications in the TSA group and eight (2.4%) complications in the RSA group (P = 0.0096). The complications in the TSA group consisted of seven radiographic failures, six subscapularis failures, three posterior dislocations, two late rotator cuff insufficiencies, one arthrofibrosis, and one wound infection. The complications in the RSA group consisted of four acromial stress fractures, two base plate fractures, one periprosthetic infection, and one periprosthetic fracture.

Glenoid Type B2 Subgroup

Of our 873 glenoids, a total of 242 glenoids could be identified as Walch type B2 (126 in the TSA group and 116 in the RSA group; Table 2, column 8). The mean ages in the TSA and RSA groups were 68.20 ± 3.25 and 73.03 ± 1.49, respectively (P = 0.25). The percentage of males in the TSA and RSA groups was 74.6% and 46.5%, respectively (P = 0.0003). The results regarding ASES scores, SANE scores, forward elevation, and external rotation are summarized in Table 6.

**Table 6:** Descriptive summary of the available data from our included studies regarding the type B2 glenoid. Asterisk (*) indicates that this data was not included in the weighted mean difference values given the lack of corresponding pre-operative data. Plus sign (+) indicates that this data was provided by the authors as mean differences as opposed to preoperative and postoperative data. TSA: total shoulder arthroplasty, RSA: reverse shoulder arthroplasty

TSA or RSA	Lead author last name	Walch type B2 shoulder number	Preoperative ASES scores	Postoperative ASES scores	Preoperative SANE scores	Postoperative SANE scores	Preoperative forward elevation	Postoperative forward elevation	Preoperative external rotation	Postoperative external rotation	
TSA	Alentorn-Geli [9]	15	Not available	91.2 ± 6.7^*^	Not available	Not available	87.3 ± 22.4	164.9 ± 13.8	9.6 ± 24.9	61.7 ± 19.2	
TSA	Sheth [15]	111	39.8 ± 18.2	88.8 ± 18.6	30.3 ± 22.1	76.0 ± 33.5	Not available	Not available	10.7 ± 14.3	45.5 ± 12.1	
RSA	Alentorn-Geli [9]	16	Not available	80.3 ±14.3^*^	Not available	Not available	86.9 ± 18.9	160.0 ± 22.5	10.0 ± 13.1	53.7 ± 34.4	
RSA	Waterman [19]	43	49.2 ± 25.3^+^		71.8 ± 44.9^+^		60.0 ± 56.8^+^		30.8 ± 38.0^+^		
											RSA	Pettit [20]	57	36.1 ± 17.0	88.8 ± 13.2	30.1 ± 19.0	91.9 ± 13.9	90.4 ± 21.0	140.1 ± 15.4	24.9 ± 10.7	56.2 ± 17.9	
Average mean difference (TSA)			49		45.7		77.6		38.6		
Average mean difference (RSA)			51.2		66.1		58.6		34.1		

Discussion

Our results demonstrated that TSA and RSA perform equally well in the setting of PGHO with an intact rotator cuff regarding forward flexion, external rotation in adduction, internal rotation, VAS-Pain scores, ASES scores, and SANE scores. There is a growing body of literature favoring the use of the RSA in many end-stage shoulder pathologies. Coscia et al. (2021) demonstrated no significant differences in the postoperative range of motion, ASES scores, and Constant-Murley scores after RSA in the treatment of massive cuff tears without osteoarthritis, cuff tear arthropathy, proximal humerus fractures, and PGHO [5]. As the RSA continues to evolve, indications have continued to expand with improved clinical results. Heifner et al. (2021) evaluated the use of RSA in PGHO [23]. These authors demonstrated an average postoperative ASES score of 77.8 and a revision rate of 3.1% after RSA in the setting of PGHO. This lends credibility to the use of the RSA in the treatment of PGHO but does not evaluate the optimal surgical solution in the patient with an intact rotator cuff or with a type B2 glenoid.

Kim et al. evaluated the TSA and the RSA in patients with PGHO and also found no difference concerning patient-reported outcomes [24]. However, these authors found a difference in external rotation and did not find a difference in terms of revision rate. There are, however, significant differences in methodology to consider when interpreting our differing results. In particular, Kim et al. included level IV evidence, only included articles with a direct comparison between RSA and TSA, and included articles without preoperative data [24]. In our study, only articles with preoperative and postoperative data were included in order to compare mean differences as the primary outcome measure as opposed to postoperative scores alone. It is important to consider these different methodologies when interpreting the difference in results from our study and the important work done by Kim et al. [24].

An interesting aspect of our clinical question is with regard to the management of glenoid bone deformity. In this setting, recent literature may favor the use of reverse shoulder arthroplasty. However, a variety of surgical options exist for the management of extensive glenoid bone deformity when using TSA including eccentric reaming [17], bone grafting [25], posteriorly augmented glenoid components [26,27], and the anterior-offset humeral head [28]. The current evidence does not seem to as strongly suggest the need for bone grafting severe glenoid bone deformity during an RSA, though the studies on this topic are limited [1].

Currently, it is difficult for shoulder surgeons to use the Walch glenoid type to the decision between TSA and RSA. In our study, there was a statistically significantly higher proportion of males in the TSA group (73.2%) as compared to the RSA group (51.1%). This may be multifactorial including gender variations in cuff atrophy and surgeon bias. However, preoperative range of motion and preoperative patient-reported outcomes did not differ significantly between the groups. Alentorn-Geli et al. quantified glenoid retroversion and posterior subluxation in the setting of the B2 glenoid between the TSA and RSA groups. They found no significant differences. In their study, they found significantly higher simple shoulder test scores and a significantly higher complication rate in the TSA group. In the present study, we also attempted to more closely look at the subgroup of patients who could be identified as Walch type B2. We demonstrated comparable changes in the ASES scores and external rotation, greater forward elevation changes in the TSA group, and greater SANE changes in the RSA group. Unfortunately, this was only possible for four of the included studies, for a total of 242 shoulders. Given the limited number of studies and heterogeneity of reported data, these results were performed solely with descriptive statistics only and thus should be interpreted with caution.

An important additional consideration in surgical decision-making is the longevity of the implants and revision options dictated by each surgical option. TSA lends the advantage of conversion to an RSA as an option in the future. However, the options in the setting of a primary RSA are revision RSA with further bone loss. This needs to be considered in the context of the current longevity data. A recent systematic review demonstrated l0-year survival rates of 94.6% and 94.4% for TSA and RSA in the context of PGHO, respectively [29]. In the context of equivalent survival rates, surgeons should give due consideration to the limitation of revision options for a primary RSA.

The results of the present study may suggest that both TSA and RSA may perform equally well in the setting of primary glenohumeral osteoarthritis with an intact cuff. However, when considering the ease of surgery and complexity of glenoid bone loss, the RSA may be the implant of choice in many scenarios. This may hold true in the setting of the type B2 glenoid as well; however, our limited subgroup in our study is insufficient to comment on this. Overall, when looking at PGHO with an intact rotator cuff, the RSA may be considered non-inferior to the TSA.

Limitations

Many of the studies included in this systematic review were retrospective in nature, which introduces a risk of confounding by surgical indication. There was an inhomogeneity of our groups with the group undergoing RSA being significantly older than those undergoing TSA. There was heterogeneity in terms of reported data with I_2_ statistics greater than 50% in most of our outcome measures, and thus the random effects model as opposed to a fixed effects model was chosen. Furthermore, ASES scores, forward flexion, and external rotation were reported in almost all studies, but the remaining outcome measures were not unanimously seen, and this should be considered when interpreting those results (IR, VAS, and SANE scores). Lastly, although we do descriptively summarize the performance of TSA and RSA in the included patients with a type B2 glenoid, ultimately, the patient number included in this subgroup was small, and we were not able to perform meaningful statistics with regard to the type B2 glenoid. Future research studies should aim to address this issue by separating the subjects into Walch B1 and Walch B2 groups to better understand the relationship between subluxation and retroversion on the performance of TSA and RSA.

## Conclusions

In the setting of PGHO with an intact rotator cuff, the RSA has a similar range of motion and clinical outcomes, but lower complication and revision rates as compared to TSA. This may hold true in the setting of the B2 glenoid, although a high-powered study on this subgroup is required. Anatomic shoulder arthroplasty maintains an important role in select patients. Further studies are required to better elucidate the role of glenoid bone loss and posterior humeral head subluxation with regard to implant choice.
